# Supplementary material for: Little evidence of inbreeding depression for birth mass, survival and growth in Antarctic fur seal pups
Source: Sci Rep. 2024 Jun 1;14:12610. doi: 10.1038/s41598-024-62290-x (PMC11144264; doi:10.1038/s41598-024-62290-x)
Supplement: Supplementary file 2 — Supplementary Information 2. [file 41598_2024_62290_MOESM2_ESM.pdf]

Supplementary information

Little evidence of inbreeding depression for birth mass, survival and growth in Antarctic fur seal pups

Anneke J. Paijmans, Ane Liv Berthelsen, Rebecca Nagel, Felicitas Christaller, Nicole Kröcker, Jaume Forcada, and Joseph I. Hoffman

Supplementary Figures

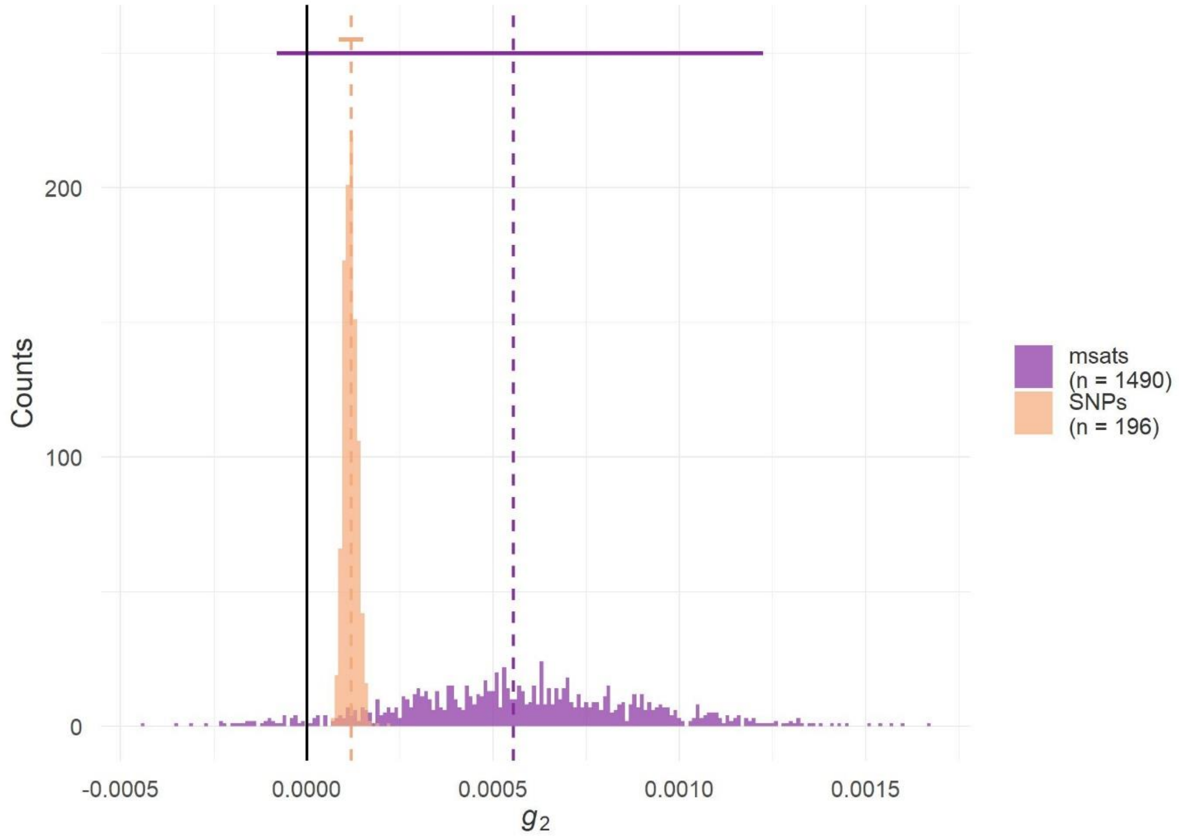

Figure S1. Distribution of identity disequilibrium estimates for both microsatellite data (purple, 1,490 individuals, 39 loci) and SNP array data (orange, 196 individuals, 75,101 autosomal variants). Estimates were based on 1,000 bootstrap replicates and 1,000 permutations. The vertical dashed lines show the empirical g_2 estimates and the horizontal bars show the 95% confidence intervals for each dataset. For both datasets, the empirical g_2 was significantly larger than zero (microsatellite data: $p = 0.028$, SNP array data: $p = 0.001$).

Supplementary Tables

Table S1. Parameter estimates from the linear models testing for the effect of individual and maternal heterozygosity (sMLH) on pup birth mass (a) including maternal genetic diversity and mother age; and (b) excluding maternal effects. Estimates are shown together with confidence intervals (CI), significant *p*-values are in bold. For both models, total number of observations, as well as the variance explained by the predictors (R^2) and variance adjusted for the number of predictors (R^2 adjusted) are reported.

Pup birth mass

	**(a) model incl. maternal effect**				**(b) model excl. maternal effect**			
Predictors	*Estimates*	*CI*	*t value*	*p*	*Estimates*	*CI*	*t value*	*p*
Intercept	4.27	3.47 – 5.07	10.52	**<0.001**	4.50	4.04 – 4.96	19.25	**<0.001**
pup sMLH	0.01	-0.63 – 0.64	0.02	0.984	0.16	-0.29 – 0.61	0.70	0.484
pup sex [M]	0.64	0.53 – 0.75	11.34	**<0.001**	0.56	0.48 – 0.64	13.22	**<0.001**
season [2019]	-0.23	-0.39 – -0.06	-2.66	**0.008**	-0.19	-0.31 – -0.07	-3.17	**0.002**
season [2020]	0.09	-0.06 – 0.24	1.21	0.227	0.16	0.04 – 0.27	2.75	**0.006**
season [2021]	-0.13	-0.28 – 0.02	-1.76	0.079	-0.25	-0.37 – -0.14	-4.45	**<0.001**
mother sMLH	-0.00	-0.63 – 0.63	-0.00	0.997				
mother age	0.04	0.02 – 0.06	3.53	**<0.001**				
Observations	342				885			
R^2 / R^2 adjusted	0.329 / 0.315				0.211 / 0.206			

Table S2. Parameter estimates from the generalized linear models (GLM) with a binomial error distribution testing for the effect of individual and maternal heterozygosity (sMLH) on pup survival (a) including maternal genetic diversity and mother age, and (b) excluding maternal effects. Log-odd ratios are shown together with confidence intervals (CI), significant *p*-values are in bold. Total number of observations, as well as the variance explained (R^2 Tjur) are reported for both models.

Pup survival

	(a) model incl. maternal effect				(b) model excl. maternal effect			
Predictors	*Log-Odds*	*CI*	*t value*	*p*	*Log-Odds*	*CI*	*t value*	*p*
Intercept	1.05	-4.28 – 6.43	0.39	0.699	-4.92	-7.39 – -2.51	-3.96	**<0.001**
pup sMLH	-3.11	-6.77 – 0.45	-1.69	0.091	0.96	-0.98 – 2.90	0.97	0.333
pup sex [M]	-0.92	-1.66 – -0.20	-2.49	**0.013**	-0.53	-0.93 – -0.14	-2.63	**0.009**
pup birth mass	0.91	0.31 – 1.53	2.94	**0.003**	1.20	0.89 – 1.53	7.34	**<0.001**
season [2019]	0.95	-0.04 – 2.12	1.76	0.078	1.08	0.47 – 1.73	3.36	**0.001**
season [2020]	0.78	-0.09 – 1.74	1.70	0.089	0.09	-0.42 – 0.61	0.35	0.730
season [2021]	0.07	-0.67 – 0.83	0.18	0.854	-0.52	-0.98 – -0.08	-2.28	**0.022**
mother sMLH	-0.94	-4.52 – 2.58	-0.52	0.604				
mother age	0.07	-0.05 – 0.19	1.15	0.251				
Observations	342				885			
R^2 Tjur	0.070				0.132			

Table S3. Parameter estimates from the linear models testing for the effect of individual and maternal heterozygosity (sMLH) on pup growth (a) including maternal genetic diversity and mother age, and (b) excluding maternal effects. Estimates are shown together with confidence intervals (CI), significant *p*-values are in bold. For both models, total number of observations, as well as the variance explained by the predictors (R^2) and variance adjusted for the number of predictors (R^2 adjusted) are reported.

Pup growth

	**(a) model incl. maternal effect**				**(b) model excl. maternal effect**			
Predictors	*Estimates*	*CI*	*t value*	*p*	*Estimates*	*CI*	*t value*	*p*
Intercept	-1.37	-3.91 – 1.17	-1.06	0.289	-1.50	-2.93 – -0.06	-2.05	**0.041**
pup sMLH	1.98	0.29 – 3.67	2.31	**0.022**	0.19	-0.93 – 1.31	0.33	0.739
pup sex [M]	0.43	0.06 – 0.79	2.31	**0.021**	0.47	0.24 – 0.70	3.98	**<0.001**
pup birth mass	-0.01	-0.31 – 0.29	-0.07	0.942	0.28	0.10 – 0.45	3.06	**0.002**
pup age	0.05	0.04 – 0.07	5.73	**<0.001**	0.06	0.05 – 0.07	10.57	**<0.001**
season [2019]	1.67	1.22 – 2.12	7.26	**<0.001**	1.75	1.46 – 2.04	11.93	**<0.001**
season [2020]	2.02	1.62 – 2.43	9.81	**<0.001**	1.62	1.33 – 1.91	11.00	**<0.001**
season [2021]	1.51	1.10 – 1.93	7.15	**<0.001**	1.39	1.10 – 1.69	9.20	**<0.001**
mother sMLH	-0.80	-2.45 – 0.85	-0.95	0.341				
mother age	0.03	-0.04 – 0.09	0.85	0.397				
Observations	291				722			
R^2 / R^2 adjusted	0.450 / 0.433				0.389 / 0.383			

Table S4. Parameter estimates from the linear model testing for the effect of individual and maternal inbreeding (F_{ROH}) on pup birth mass. Estimates are shown together with confidence intervals (CI), significant *p*-values are in bold. Total number of observations, as well as the variance explained by the predictors (R^2) and variance adjusted for the number of predictors (R^2 adjusted) are given.

Pup birth mass

	**model incl. maternal effect**			
Predictors	*Estimates*	*CI*	*t value*	*p*
Intercept	4.69	3.01 – 6.37	5.54	**<0.001**
pup F_{ROH}	3.37	-12.91 – 19.64	0.41	0.682
mother F_{ROH}	3.77	-11.56 – 19.10	0.49	0.626
pup sex [M]	0.44	0.07 – 0.81	2.35	**0.021**
season [2020]	0.28	-0.07 – 0.63	1.59	0.116
colony [SSB]	0.12	-0.24 – 0.47	0.66	0.510
Observations	91			
R^2 / R^2 adjusted	0.092 / 0.038			

Table S5. Parameter estimates from the generalized linear model (GLM) with a binomial error distribution testing for the effect of individual and maternal inbreeding (F_{ROH}) on pup survival. Estimates are shown together with confidence intervals (CI), significant *p*-values are in bold. Total number of observations, as well as the variance explained (R^2 Tjur) are given.

Pup survival

	**model incl. maternal effect**			
Predictors	*Log-Odds*	*CI*	*t value*	*p*
Intercept	-4.22	-11.31 – 2.24	-1.24	0.216
pup F_{ROH}	28.96	-21.78 – 83.83	1.09	0.277
mother F_{ROH}	-19.35	-73.54 – 32.49	-0.72	0.469
pup sex [M]	0.11	-1.21 – 1.40	0.17	0.867
season [2020]	-0.13	-1.30 – 1.01	-0.23	0.821
colony [SSB]	1.36	0.20 – 2.68	2.19	**0.028**
pup birth mass	0.80	0.09 – 1.65	2.04	**0.042**
Observations	91			
R^2 Tjur	0.153			

Table S6. Parameter estimates from the linear model testing for the effect of individual and maternal inbreeding (F_{ROH}) on growth. Estimates are shown together with confidence intervals (CI), significant *p*-values are in bold. Total number of observations, as well as the variance explained by the predictors (R^2) and variance adjusted for the number of predictors (R^2 adjusted) are given.

Pup growth

	**model incl. maternal effect**			
Predictors	*Estimates*	*CI*	*t value*	*p*
Intercept	-7.59	-13.20 – -1.99	-2.71	**0.009**
pup F_{ROH}	-4.30	-39.31 – 30.71	-0.25	0.807
mother F_{ROH}	47.33	16.61 – 78.06	3.08	**0.003**
pup sex [M]	2.10	1.33 – 2.87	5.45	**<0.001**
season [2020]	-0.45	-1.18 – 0.29	-1.22	0.227
colony [SSB]	-0.13	-0.83 – 0.57	-0.37	0.712
pup birth mass	0.15	-0.33 – 0.62	0.62	0.535
pup age	0.13	0.08 – 0.18	4.88	**<0.001**
Observations	72			
R^2 / R^2 adjusted	0.444 / 0.384			
